# Single-cell multi-omics reveals the TNF-α activation threshold for Classical Monocytes by studying healthy donors and rheumatoid arthritis patients

**DOI:** 10.3389/fimmu.2025.1572823

**Published:** 2025-05-16

**Authors:** Roman Perik-Zavodskii, Olga Perik-Zavodskaia, Saleh Alrhmoun, Julia Lopatnikova, Alina Alshevskaya, Julia Zhukova, Julia Shevchenko, Nadezhda Shkaruba, Natalia Sivitskaya, Shakir Suleimanov, Elizaveta Sheveleva, Kirill Nazarov, Fedor Kireev, Alexey Sizikov, Elena Golikova, Sergey Sennikov

**Affiliations:** ^1^ Laboratory of Molecular Immunology, Federal State Budgetary Scientific Institution Research Institute of Fundamental and Clinical Immunology, Novosibirsk, Russia; ^2^ Laboratory of Immune Engineering, Federal State Autonomous Educational Institution of Higher Education I.M. Sechenov First Moscow State Medical University of the Ministry of Health of the Russian Federation (Sechenov University), Moscow, Russia; ^3^ Rheumatology Department of the Immunopathology Clinic, Federal State Budgetary Scientific Institution Research Institute of Fundamental and Clinical Immunology, Novosibirsk, Russia

**Keywords:** TNF-α, TNFR1, TNFR2, response to TNF-α, rheumatoid arthritis, CITE-seq, ScRNA-seq

## Abstract

**Introduction:**

Tumor Necrosis Factor Alpha is a known pro-inflammatory cytokine that plays a key role in the pathogenesis of rheumatoid arthritis. Anti-cytokine therapies targeting Tumor Necrosis Factor Alpha have greatly succeeded in treating rheumatoid arthritis in many patients. Despite these developments, many of the mechanisms of Tumor Necrosis Factor Alpha action have yet to be uncovered.

**Methods:**

In this study, we incubated PBMCs from healthy donors and rheumatoid arthritis patients with Tumor Necrosis Factor Alpha and then performed their single-cell multi-omics analysis via BD Rhapsody.

**Results:**

We have observed that Classical Monocytes have responded to the Tumor Necrosis Factor Alpha stimulation the most and that there was an activational threshold for such response that was dependent on the TNFR2 protein expression level.

**Discussion:**

The profiling of TNFR2 protein expression level on immune cell populations can be a good predictive factor for the assessment of their activation by Tumor Necrosis Factor Alpha.

## 1 Introduction

Rheumatoid Arthritis (RA) is a chronic autoimmune disease that primarily affects synovial joints, leading to persistent inflammation, progressive cartilage degradation, and bone erosion (1–3). The global prevalence of RA is estimated to be around 0.5–1% in the general adult population, making it one of the most common inflammatory arthritis conditions worldwide (4, 5). Despite advances in treatment strategies, many patients continue to experience active disease and joint damage, emphasizing the need for a deeper understanding of RA pathogenesis to optimize therapeutic interventions (6).

A hallmark of RA is the dysregulated immune response within the synovium. Multiple immune system components, such as T cells, B cells, macrophages, and dendritic cells are involved in the pathogenesis of RA (7–9). Auto-reactive T and B cells contribute to forming immune complexes, stimulating the synovial membrane and infiltrating leukocytes to release pro-inflammatory cytokines and chemokines (10, 11). These events maintain the progression of local joint inflammation and can also have systemic consequences, that indicate that the pathogenesis of the disease can extend beyond the joint. Thus, the composition of immune cell subtypes in RA may differ from normal conditions and reflect the disease progression.

Among the wide spectrum of inflammatory mediators, tumor necrosis factor (TNF) plays a particularly central role in RA pathogenesis (12, 13). TNF is a central pro-inflammatory cytokine that orchestrates a wide range of immune responses, particularly those involving cell-mediated immunity (14). Primarily produced by activated macrophages and T lymphocytes, TNF exerts pleiotropic effects on various immune cells, helping regulate the balance between protective inflammation and pathological damage (15). Under physiological conditions, TNF is critical for immune surveillance, promoting the clearance of pathogens and tumor cells (16). However, excessive TNF production can trigger a self-perpetuating cycle of inflammation, driving the pathophysiological processes seen in autoimmune disorders such as rheumatoid arthritis (RA) in which elevated levels of TNF in the synovium and serum correlate with disease activity and joint damage (17, 18).

Two main types of receptors mediate the effects of TNF on immune cells: TNFR1 and TNFR2. TNFR1 is expressed on nearly all cell types, whereas TNFR2 is found on the surface of select cell populations, including immune cells (19). Importantly, TNFR2 is predominantly expressed on immune cells and is primarily activated by the membrane-bound form of TNF. In contrast, TNFR1 is activated by both the membrane-bound and soluble forms of the ligand (20). The downstream effects of TNFR1 activation vary significantly, ranging from NF-κB activation with subsequent activation of gene expression and survival to the initiation of apoptosis and necroptosis (21). In contrast, TNFR2 predominantly activates both canonical and non-canonical NF-κB pathways. Canonical NF-κB activation leads to the rapid expression of pro-inflammatory genes, whereas non-canonical NF-κB activation supports cell survival and proliferation over a slower timescale (22). Thus, the balance of TNFR1 and TNFR2 expression on immune cells plays a critical role in finely tuning the physiological state of the cell.

TNF blockade has revolutionized RA treatment over the past decades. By neutralizing TNF activity, disease-modifying antirheumatic drugs (DMARDs) can reduce synovitis, slow the progression of joint damage, and improve clinical outcomes. However, primary or secondary nonresponse to TNF inhibitors still occurs in a subset of patients, highlighting the intricate network of additional pathways that drive RA pathology and the need to explore new therapeutic targets (12, 23).

**Figure 1:**
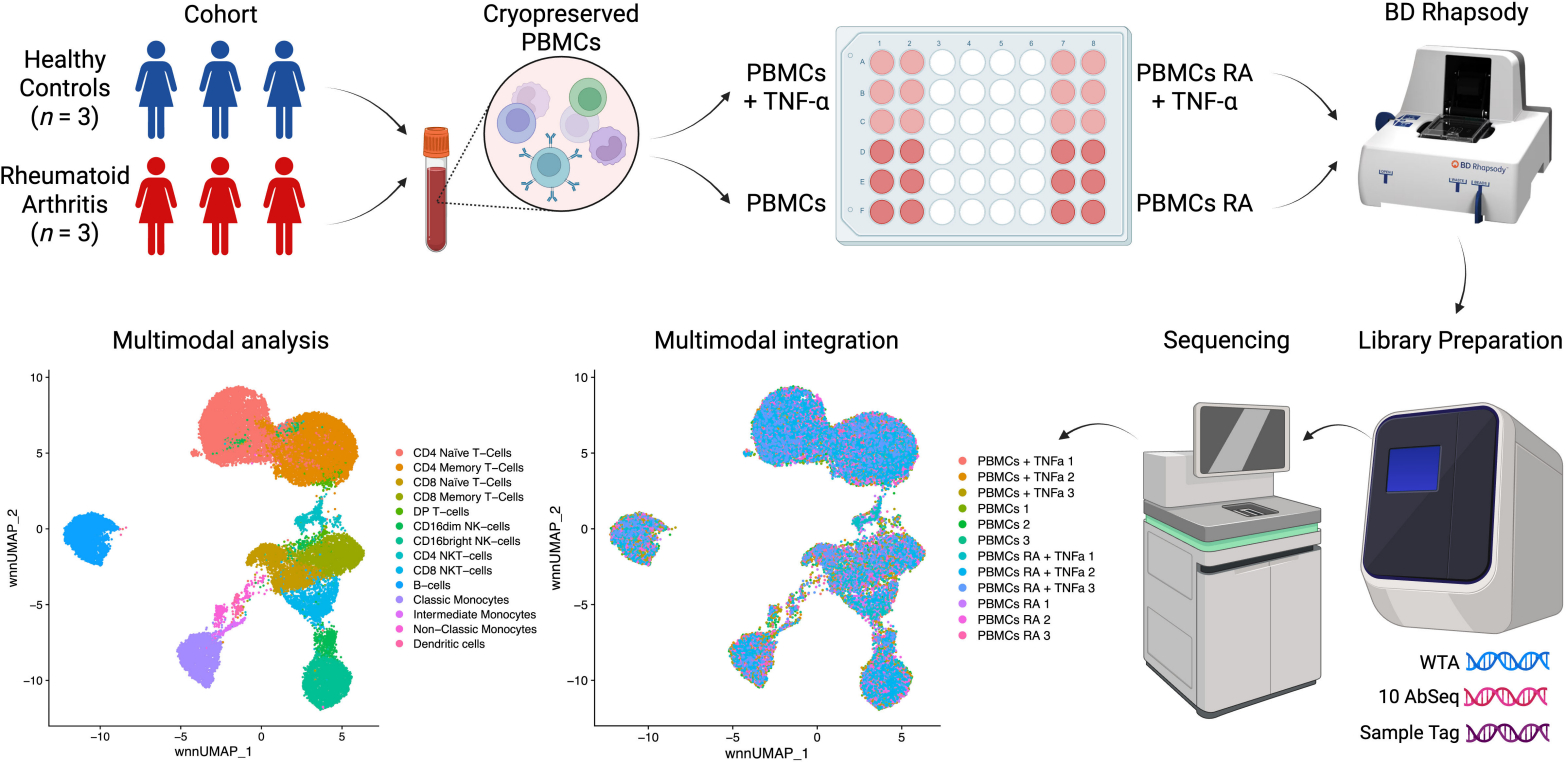
Overview of the experiment. This figure was created via BioRender.

Recent advancements in single-cell sequencing have led to significant discoveries in immunology (24–27), providing powerful tools to analyze complex immune responses at high resolution. Leveraging this technology, our study investigates transcriptional profiles in RA and healthy controls, focusing on the impact of ligand stimulation of TNFR1 and TNFR2 surface expression. Using the BD Rhapsody platform and the Cellular Indexing of Transcriptomes and Epitopes (CITE-seq) method, we simultaneously analyze whole transcriptome (WTA) and surface protein expression (10 surface proteins) in peripheral blood mononuclear cells (PBMCs), allowing us to capture transcriptional changes associated with receptor activation (**Figure 1**).

## 2 Materials and methods

### 2.1 Activation of PBMCs by TNF

#### 2.1.1 Collection of clinical material

We obtained peripheral blood from healthy donors and patients diagnosed with rheumatoid arthritis (RA). After signing an informed consent form, RA patients were recruited at the Clinic of Immunopathology of the Research Institute for Fundamental and Clinical Immunology. The study included patients with rheumatoid arthritis (*n* = 3, women, 57–67 years old), who had a high degree of disease activity (DAS28 ranging 5.17-6.69), seropositive for rheumatoid factor and positive for antibodies to cyclic citrullinated peptide, receiving basic therapy with Methotrexate or Leflunomide, and different total duration of the disease. Subsequently, we recruited a group of conditionally healthy donors (*n* = 3, women, 62–68 years old), comparable in gender and age and without rheumatological pathologies.

#### 2.1.2 Isolation and cultivation of PBMCs

We collected blood (up to 9 mL) from the cubital vein under sterile conditions and in the fasting state into vacuum tubes containing the anticoagulant K3-EDTA (the tripotassium salt of ethylenediaminetetraacetic acid; Vacuette K3-EDTA, Greiner Bio-One GmbH, Austria). Before the experiment, we prepared a complete culture medium by supplementing RPMI with 10% fetal bovine serum (HyClone, Logan, UT, USA), 2 mM L-glutamine (BioloT, St. Petersburg, Russia), 5 × 10^−4 M 2-mercaptoethanol (Sigma-Aldrich, St. Louis, MO, USA), 80 µg/mL gentamicin (KRKA, Novo mesto, Slovenia), 10 mM HEPES (Sigma-Aldrich), and 100 µg/mL benzylpenicillin (Biosintez, Russia). We isolated peripheral blood mononuclear cells (PBMCs) using a Ficoll-Urografin gradient (1.077 g/mL, PanEco, Russia). We then cryopreserved PBMCs immediately after isolation in a solution containing 90% fetal bovine serum and 10% DMSO for subsequent single-cell multiomics analysis.

#### 2.1.3 PBMC incubation with TNF

To study the effects of TNF on PBMCs, we unfroze the PBMCs, prepared two aliquots, and then incubated one of the aliquots (5.0 × 10^5^ cells) with TNF at the concentration of 5 ng/µl for 6h in the complete RPMI culture medium.

### 2.2 Single-cell multi-omics analysis

#### 2.2.1 Sample Tag and AbSeq cell staining and counting

After the TNF incubation we incubated mononuclear cells with Sample Tag (1–6) antibodies from the BD™ Single-Cell Multiplexing Kit (633781, BD Biosciences, San Jose, CA, USA) to barcode individual samples and 10 AbSeq (CD4:SK3 | CD4 | AHS0032 | Cat#940001, CD8:SK1 | CD8A | AHS0228 | Cat#940305, CD14:MPHIP9 | CD14 | AHS0037 | Cat#940005, CD16:B73.1 | FCGR3A_FCGR3B | AHS0242 | Cat#940314, CD19:HIB19 | CD19 | AHS0161 | Cat#940247, CD45RA: HI100 | PTPRC | AHS0009 | Cat#940011, CD45RO | PTPRC | AHS0036 | Cat#940022, CD56:NCAM16.2 | NCAM1 | AHS0019 | Cat#940007, CD120A (TNFR1) | TNFRSF1A | AHS0439 | 46033, CD120B (TNFR2) | TNFRSF1B | AHS0421 | 460318, BD Biosciences) antibodies for surface protein expression profiling for 30 minutes at room temperature according to the manufacturer’s recommendations (“Single Cell Labelling with BD AbSeq Ab-Oligos (1 to 40 plex)”).

After three washing cycles, cells were stained with Calcein according to the BD Rhapsody Single-Cell Analysis System User Guide. Calcein-positive cells were counted using the Attune NxT flow cytometer as events/uL. Cells were then pooled together in equal proportions and resuspended in a cold sample buffer to a final concentration of 30 cells/µl for loading onto a BD Rhapsody Cartridge. The number of cells loaded into the cartridge was visually validated using the In Cell Analyzer 6000 as mean Calcein-positive cells in 5 fields of view (FOV)/175 (microwells per FOV) * 200000 (total number of microwells per cartridge). Healthy donors’ (*n* = 3) and rheumatoid arthritis patients’ (*n* = 3) PBMCs were loaded onto two separate BD Rhapsody cartridges within 30 minutes of each other and then were further processed simultaneously to avoid any additional batch effect.

#### 2.2.2 CITE-seq library preparation and sequencing

We performed single-cell capture and cDNA library preparation using the BD Rhapsody Express Single-Cell Analysis System (BD Biosciences), according to the manufacturer’s instructions (Whole Transcriptome Analysis (WTA), Sample Tag, and BD AbSeq Library Preparation Protocol). Briefly, we captured single cells in the BD Rhapsody cartridge, added magnetic beads for poly-A-based mRNA capture, along with Sample Tag and AbSeq, lysed the cells, performed reverse transcription of the poly-A captured mRNA, AbSeq and Sample Tag on the magnetic beads, treated the beads with Exonuclease I, denatured the Sample Tag and AbSeq from the beads, performed Sample Tag and AbSeq PCR1, performed RPE (Random Priming and Extension) on the beads with cDNA, denatured and collected the RPE product, and performed another round of RPE, performed single-sided cleanup of the RPE product using AMPure XP Beads (A63880, Beckman Coulter, Brea, CA, USA), to remove primer dimers and other small molecular weight by-products. Following that, we further amplified the purified RPE product and purified the resulting RPE PCR (WTA) and Sample Tag and AbSeq PCR1 products using single-sided selection with the help of AMPure XP Beads. We then assessed their concentrations with the Qubit 4 Fluorometer and the Qubit dsDNA High-Sensitivity Assay Kit (Q32854, Thermo Fisher, Waltham, MA, USA) and performed quality control using Qsep1 capillary electrophoresis with the S2 Cartridge (Bioptic, China). After that, we further amplified the Sample Tag PCR1 product and purified the resulting PCR2 product using single-sided selection with AMPure XP Beads, performed a final round of amplification using indexes for Illumina sequencer to prepare the final libraries for the WTA and Sample Tag PCR2 products, as for the AbSeq PCR1 product, we normalized its concentration to 1 ng/μL and then performed index PCR. Eventually, we performed library clean-up using single-sided selection with AMPure XP Beads for the Sample Tag and AbSeq index libraries, and double-sided selection for the WTA index library. Library concentrations were then assessed using the Qubit 4 Fluorometer with the Qubit dsDNA High-Sensitivity Assay Kit, followed by quality control with Qsep1 capillary electrophoresis and the S2 Cartridge. We then pooled the final libraries (~82/16/2% WTA/AbSeq/Sample Tag ratio, estimated read/cell: 50000 (WTA), 10000 (AbSeq, 1000 reads per AbSeq) and 1200 (Sample Tag)) to the final concentration of 5 nM. The final pooled libraries were sequenced (R1 = 71, R2 = 51, 1300 million clusters, S1 flow cell, one flow cell for each BD Rhapsody cartridge) on a NovaSeq 6000 sequencer (Illumina, San Diego, California, United States).

#### 2.3.3 Raw data processing

We processed the FASTQ files obtained from sequencing using the BD Rhapsody pipeline v2.0 (BD Biosciences). The pipeline removed read pairs with low quality based on their read length, mean base quality score, and highest single-nucleotide frequency, analyzed remaining high-quality R1 reads to identify cell label and unique molecular identifier (UMI) sequences, aligned WTA R2 reads to the transcriptome reference via STAR, then aligned the remaining high-quality R2 reads to AbSeq panel reference using Bowtie2, collapsed reads with the same cell label, the same UMI sequence and the same gene into a single molecule, adjusted the obtained counts by error correction algorithms, namely, recursive substitution error correction (RSEC) (WTA and AbSeq) and distribution-based error correction (DBEC) (AbSeq only) to correct for sequencing and PCR errors, estimated cell counts using the second derivative analysis to filter out noise cell labels, observed one inflection point, and considered cell labels after that point to be noise labels. Then, the pipeline used molecular barcoded oligo-conjugated Sample Tag antibodies (Single-cell multiplexing kit HS, BD Biosciences) to demultiplex the samples and filter out the cell multiplets. The pipeline called 31268 single cells (2500–3500 cells per sample) across the two BD Rhapsody cartridges and output combined gene and surface protein expression matrices for each sample. Sequencing metrics showed sequencing saturation of 92-98% and a mean RSEC sequencing depth of 5.5, which is considered above medium sequencing depth for BD Rhapsody libraries (RSEC = 1 - shallow sequencing, RSEC = 8 - deep sequencing).

#### 2.2.4 Multi-omics data analysis via Seurat WNN

We analyzed gene and surface protein expression using Seurat WNN (Weighted Nearest Neighbors) (28). We imported gene expression matrices, created Seurat objects for each sample, added AbSeq surface protein expression data to each object as ADT (antibody-derived tag) data, merged the individual objects, and subjected them to a quality control procedure (nCount_RNA < 7500, nCount_ADT < 7500, percent.mt < 23.5). The merged gene expression matrix was normalized using the SCTransform v2 (SCT) package (29) of the R programming language. For the SCT-normalized gene expression matrix, we performed PCA (principal component analysis) dimensionality reduction and corrected the batch effect using the Harmony package (30) of the R programming language. The merged ADT matrix was normalized using the Centered Log-ratio (CLR) normalization method taking into account all 10 surface proteins. For the CLR-normalized ADT matrix, we performed PCA dimensionality reduction and corrected the batch effect using the Harmony package of the R programming language. We then performed Weighted Nearest Neighbors Uniform Manifold Approximation and Projection (WNN UMAP) multi-omics dimensionality reduction using 30 Harmony-corrected gene expression principal components and 9 Harmony-corrected ADT principal components, found multi-omics neighbors and clusters (resolution = 0.7). We then manually annotated the resulting clusters using their surface protein and gene expression data and created the *DimPlot* of the clusters, *FeaturePlot*, and *VlnPlot* of the surface protein marker expression via Seurat. We used the modified *VlnPlot* by Ming Tommy Tang, (https://divingintogeneticsandgenomics.com/post/stacked-violin-plot-for-visualizing-single-cell-data-in-seurat/, accessed on 27.01.2025).

#### 2.2.5 TNF response analysis

To find single cells that TNF activated, we used AUCell (31) with the following signature response to TNF via TNFR1 genes: *IL1A, IL1B, CCL3, CCL4, CXCL2, CXCL3, SNX9, NIBAN1, JUNB, JUN, FOS, ATP2B1, CRIM1, PTGS2, TNIP3, EHD1, ID2, NBN, PSTPIP2, AK4, DRAM1, MAILR, GCH1, SNX10, MAP3K8, MTF1, MMP14, SGPP2, ACSL1, TNFAIP6, TNFAIP8* (32–35). In brief, AUCell ranked genes by their expression values, calculated the Area Under the Curve (AUC) to determine whether the genes from a given gene set were enriched in the ranked gene list for each cell. A higher AUC score indicated that a larger proportion of the gene set was highly expressed in a given cell, suggesting active expression of that gene program. We then exported TNFR1 and TNFR2 normalized protein expression, cell clustering data, and AUCell TNF activation scores to perform Pearson correlation via the *corr* function of the Pandas library (36). We represented the correlation values as a heat map via MatPlotLib (37). We also created a *DotPlot* of the TNF-activated genes, TNFR1 and TNFR2 normalized protein expression, and the AUCell TNF activation scores via Seurat for the Classical Monocytes as they had the highest AUCell TNF activation score.

Next, we exported mean TNFR1 and TNFR2 normalized protein expression and AUCell TNF activation score values via the *AverageExpression* function followed by the min-max transformation for every value to perform correlation and regression analyses for the values before and after the incubation with TNF for each sample. We used the *corr* function of the Pandas library (36) to perform Pearson correlation and MatPlotLib (37) to graph the relations between the studied factors. We then added an activation threshold for the TNFR2. The activation threshold location was detected as the intersection of TNFR2 protein expression with the midpoint of the AUC response to the TNF score that separated high TNF response levels from low or no response. The activation threshold was then added as dotted lines to the correlation graph. To convert the TNFR2 normalized protein expression back into molecule counts we fit a second-degree polynomial to the data using the predicted TNFR2 receptor counts (we assumed 1 AbSeq molecule to be equal to 1 TNFR2 receptor) for the activation threshold and created a plot via MatPlotLib (37).

#### 2.2.6 Differential gene expression analysis

To study the differences between normal and rheumatoid arthritis Classical Monocytes, we performed pseudo-bulk differential gene and TNFR1 and TNFR2 protein expression analysis via pyDeSeq2 (38) using the aggregated Classical monocyte UMI counts obtained via the *AggregateExpression* Seurat function. We used BulkOmicsTools (39) to create a volcano plot of the differentially expressed genes, we considered log2(Fold Change) > |0.847| and q-values < 0.01 significant. We then used GSEApy (40) to perform an overrepresentation analysis of the differentially expressed genes in Gene Ontology Biological Process terms.

## 3 Results

### 3.1 Multi-omics characteristics of PBMCs

**Figure 2:**
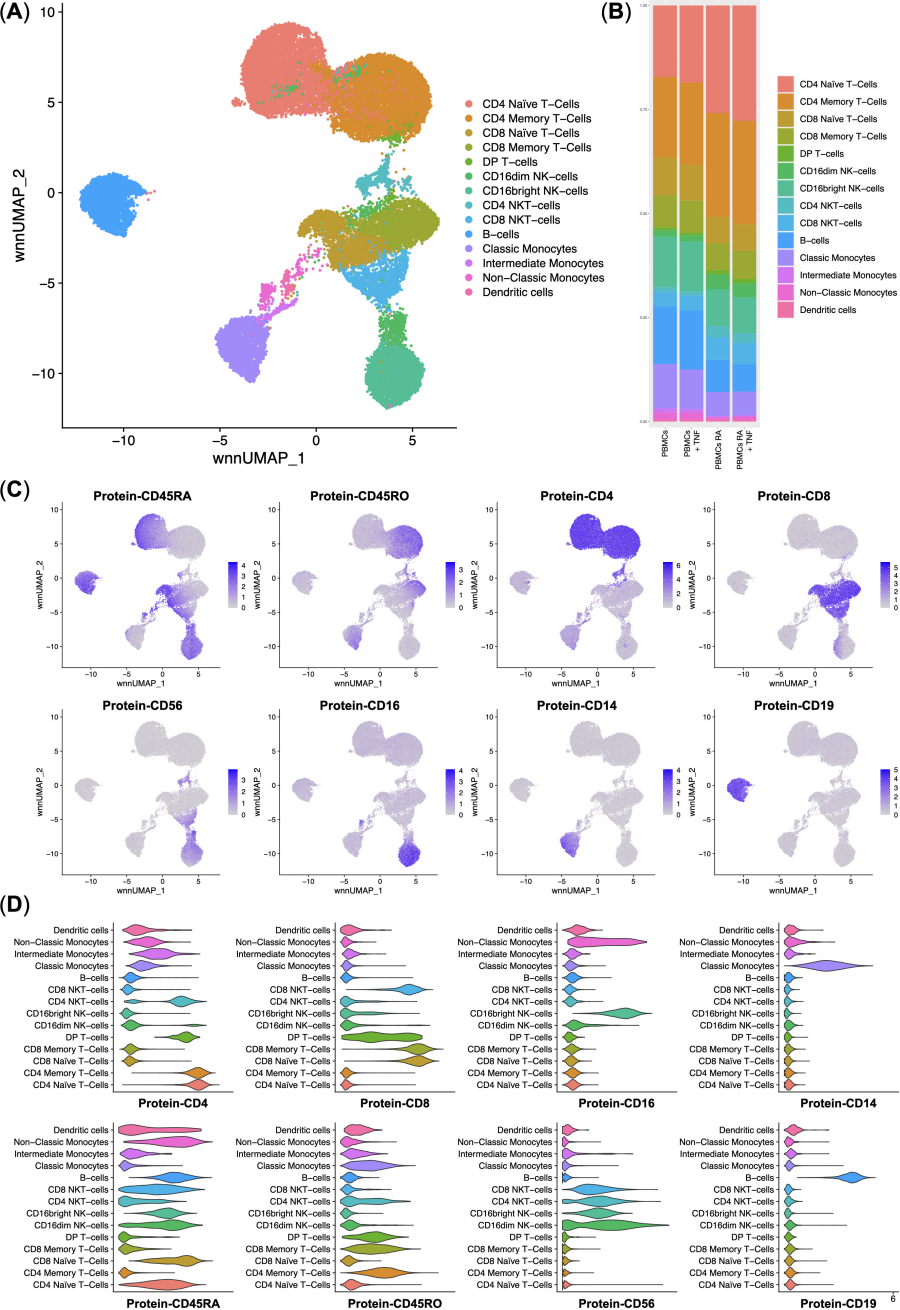
Integrated surface protein and whole transcriptome analysis of the normal (*n* = 3) and rheumatoid arthritis (*n* = 3) single peripheral blood mononuclear cells. **(A)** UMAP plot of the clusters; **(B)** Stacked bar plot of the percentages of cells per cluster per experimental group, clusters are color-labeled following the subFig A; **(C)** Feature plots of the surface protein marker expression; **(D)** Violin plots of the surface protein marker expression per cluster, clusters are color-labeled following the subFig A.

In this study, we performed a multi-omics analysis of the PBMCs from healthy donors and rheumatoid arthritis (RA) patients. First, we performed multi-omics integration and clustering of single cells. We have observed every cell population that is detectable in PBMCs: CD4 and CD8 Naïve and Memory T-cells, DP (Double-positive) T-cells, CD4 and CD8 NKT-cells, NK-cells, B-cells, Classical, Intermediate, and Non-Classical Monocytes, and Dendritic cells (**Figures 2A, C, D**). We observed that incubation of the PBMCs with TNF did not significantly disturb the cell composition and that RA patients had 12% more CD4 T-cells (**Figure 2B**).

### 3.2 TNFR2 protein expression level predicts the response to TNF via TNFR1 in classical monocytes

**Figure 3:**
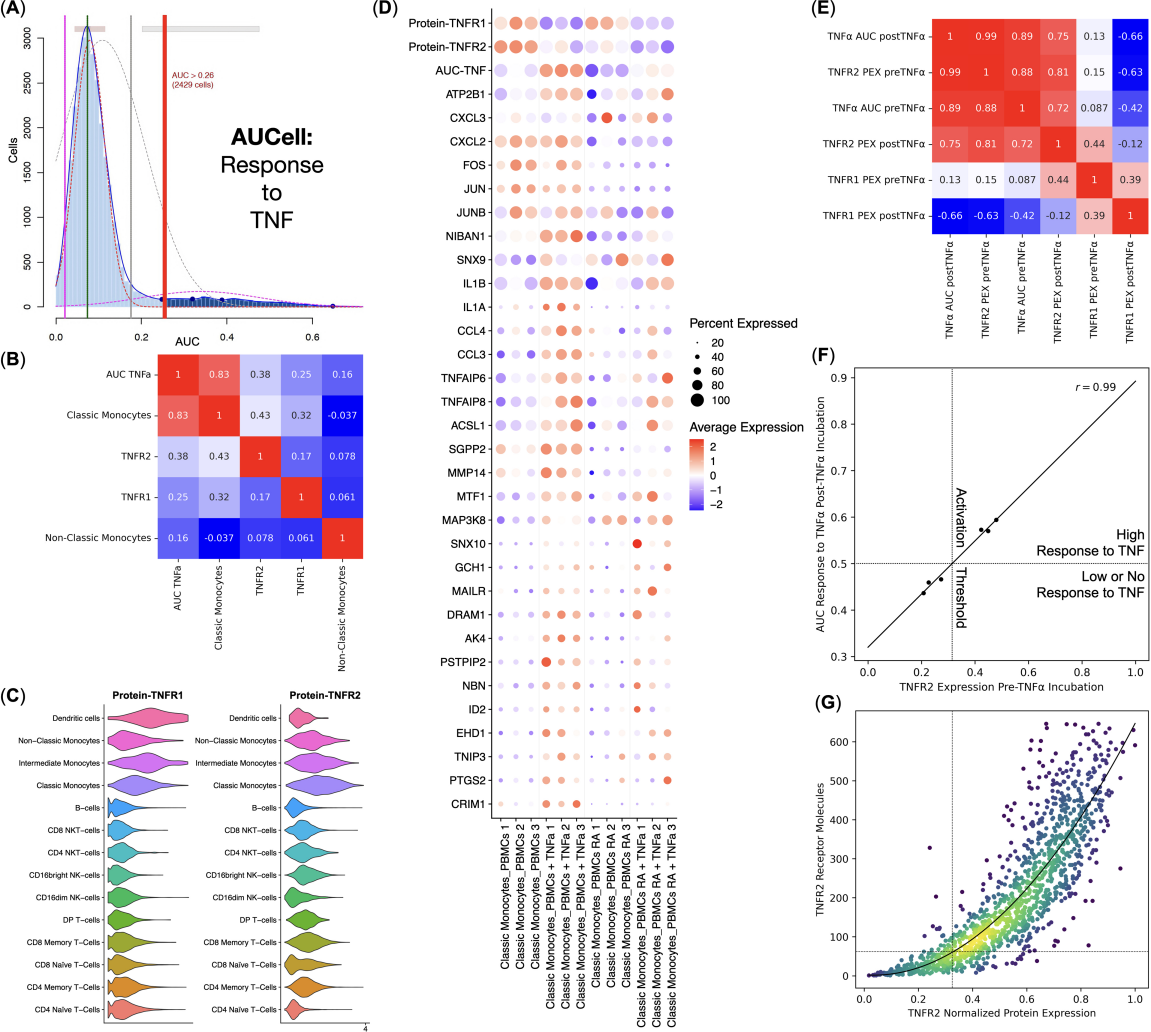
Response to TNF by the healthy donors (*n* = 3) and rheumatoid arthritis (RA) patients (*n* = 3) Classical Monocytes without the TNF stimulation. **(A)** AUCell TNF signaling pathway response detection plot, **(B)** Pearson correlation analysis of the TNF signaling pathway response activity AUCell scores, cell types, and TNFR1 and TNFR2 protein expression, **(C)** Violin plots of the TNFR1 and TNFR2 protein expression, **(D)** Dot plot of the TNF response genes, TNF response AUC scores, and TNF receptor protein expression, mean marker expression values were Z-score transformed, the blue color represents the lowest marker expression whereas the red color represents the maximum marker expression, dot size represents the percentage of Classical Monocytes positive for the marker, samples are split into biogroups by vertical dotted lines, **(E)** Pearson correlation analysis of the mean TNF signaling pathway response activity AUCell scores and normalized mean TNFR1 and TNFR2 protein expression (PEX) pre- and post TNF incubation for each sample, **(F)** linear regression of the normalized mean TNFR2 protein expression pre TNF incubation and the mean TNF signaling pathway response activity AUCell scores post TNF incubation, dotted lines depict TNF response activation threshold; **(G)** conversion between the TNFR2 normalized protein expression and the TNFR2 molecule counts, dotted lines depict the TNF response activation threshold, curve depicts the fitted second-degree polynomial.

As our main goal was to elucidate the details of the response to TNF by PBMCs via TNFR1, we used AUCell to scan for the single cells that had responded to TNF via TNFR1 using a set of signature genes (**Figure 3A**). We observed that Classical Monocytes had responded to TNF the most. TNFR2 and TNFR1 protein expression were the second and the third most important factors in response to TNF via TNFR1 (**Figure 3B**). Despite being the most activated cells through TNFR1, Classical Monocytes did not have the highest expression of the said receptor and instead had the most prominent expression of the TNFR2 among all PBMCs (**Figure 3C**).

We have also observed that normal and RA Classical Monocytes had different TNFR1 and TNFR2 protein expression, as well as a different expression of TNF response genes, and, therefore, different AUCell scores – normal Classical Monocytes had significantly higher TNFR2 protein expression and responded to TNF more prominently compared with the RA Classical Monocytes (**Figure 3D**).

We then tested the relations between TNFR1 and TNFR2 protein expression and TNF response via TNFR1 AUCell scores pre and post-TNF incubation for each sample (**Figure 3E**) and observed that TNFR2 protein expression pre-TNF incubation strongly and positively (r = 0.99) correlated with the TNF response via TNFR1 AUCell scores post-TNF incubation (**Figures 3E, F**), as well as that TNFR1 protein expression post-TNF incubation strongly and negatively (r = −0.66) correlated with the TNF response via TNFR1 AUCell scores post-TNF incubation (**Figure 3E**).

Since Classical Monocytes from healthy donors had a high response to TNF and Classical Monocytes from RA patients had a low or no response, and Classical Monocytes from healthy donors and RA patients, respectively, had high and low levels of TNFR2 protein expression before incubation with TNF (**Figure 3D**), there might be a threshold of TNFR2 protein expression that, when crossed, activates a robust response to TNF. We detected that this threshold was at 32.5% of the normalized TNFR2 protein expression, translating into 62 TNFR2 receptor molecules (**Figure 3G**).

### 3.3 Classical monocytes in rheumatoid arthritis show gene expression signature associated with foam cell differentiation

**Figure 4:**
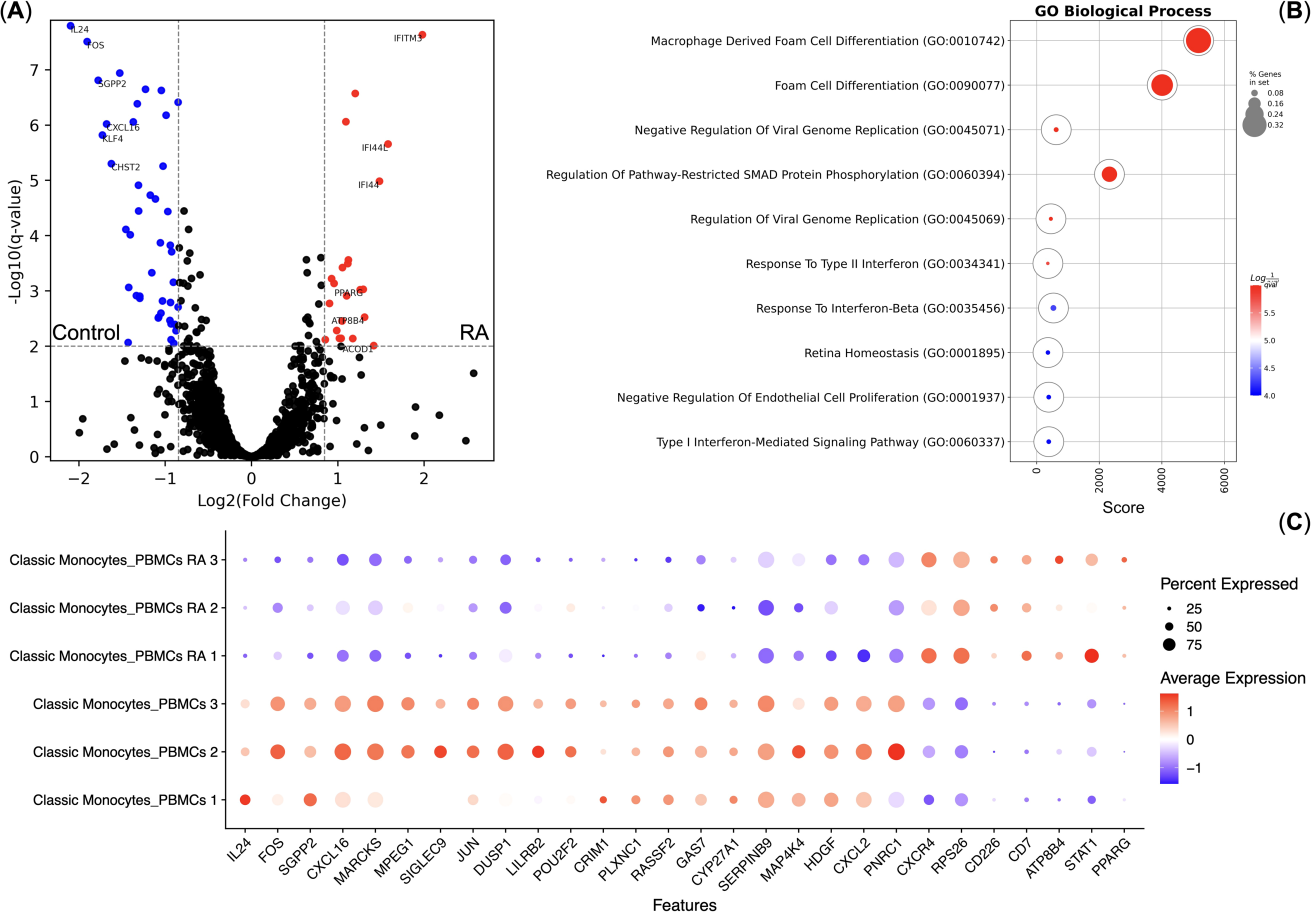
Differential gene expression analysis of the Classical Monocytes of healthy controls (*n* = 3) and rheumatoid arthritis (RA) patients (*n* = 3). **(A)** Volcano plot of the differentially expressed genes; **(B)** Gene Ontology Biological Process overrepresentation analysis of the up-regulated genes. Red corresponds to the lowest *q*-value, blue corresponds to the highest *q*-value, and the dot size reflects the percentage of genes in the analysis from the full set of genes in the Gene Ontology Biological Process database; **(C)** Dot plot of the differentially expressed genes, mean marker expression values were Z-score transformed, the blue color represents the lowest marker expression whereas the red color represents the maximum marker expression, dot size represents the percentage of Classical Monocytes positive for the marker.

As Classical Monocytes had differential responses to TNF, we also performed pseudo-bulk differential gene expression analysis between the normal and RA Classical Monocytes without the TNF stimulation. We observed that RA and normal Classical Monocytes indeed have differentially expressed genes (**Figures 4A, C**) and that genes up-regulated in the RA Classical Monocytes are enriched in the Foam cell differentiation biological process that included the *STAT1* and *PPARG* genes (**Figure 4B**). We also observed that RA Classical Monocytes had significantly higher CXCR4 chemokine receptor gene expression (**Figure 4C**).

## 4 Discussion

In this study, we performed a multi-omics analysis of the response to TNF by peripheral blood mononuclear cells of healthy donors and rheumatoid arthritis patients. We observed that Classical Monocytes responded the most to TNF, and had the most prominent TNFR2 protein expression and medium TNFR1 protein expression. Classical Monocytes from rheumatoid arthritis patients responded to TNF less prominently compared with Classical Monocytes from healthy donors. They also had an active gene expression signature of Foam cell differentiation and significantly higher *CXCR4* gene expression.

It is also worth mentioning that other immune cell populations, including T cells, exhibited no detectable response for TNF activation, suggesting that higher TNF stimulation doses may be required to elicit activation in these cells. This differential responsiveness highlights the presence of distinct regulatory mechanisms or activation thresholds governing receptor activation in different populations of immune cells, with monocytes being more sensitive to TNF stimulation than other cell types. Further research is needed to investigate these differences to gain a deeper understanding of the signaling requirements and regulatory mechanisms underlying TNF-mediated activation in diverse immune cell subsets.

Our findings support the notion that receptor expression levels play a crucial role in determining cellular responsiveness to cytokine signaling (41). The differential expression of TNFR1 and TNFR2 observed in RA and healthy monocytes suggests that distinct activation thresholds may contribute to variations in TNF-mediated signaling. Notably, we found a positive correlation between TNFR2 protein expression and the cellular response to TNF via TNFR1. This could be potentially explained by TNFR2 working as an on-cell depot for TNF and that TNF is later released from TNFR2 and is uptaken by the TNFR1 (42, 43). Additionally, the observed decrease in TNFR1 protein expression following TNF incubation was associated with a robust TNFR1-mediated response, likely due to the internalization of TNFR1 after the successful interaction with its ligand. These findings highlight the complex interplay between TNFR1 and TNFR2 in modulating TNF signaling and suggest that receptor expression dynamics play a key role in shaping immune responses.

The reduced TNFR2 expression observed in RA classical monocytes may result from chronic TNF stimulation in the disease environment, leading to receptor downregulation as a regulatory mechanism. Alternatively, RA monocytes might possess a higher activation threshold for TNFR2 signaling, requiring stronger or prolonged stimulation to elicit a comparable response to that seen in healthy monocytes.

The Foam cell formation gene expression signature in Classical Monocytes from rheumatoid arthritis patients might reflect metabolic changes occurring due to rheumatoid arthritis and validate previous similar findings (44–46). As CXCR4 is up-regulated in Classical Monocytes from rheumatoid arthritis patients, they can be expected to migrate towards CXCL12. The CXCL12-CXCR4 axis is known to be of great importance in rheumatoid arthritis (47) where abnormally high concentrations of CXCL12 in synovial fluid and overexpression of CXCL12 in synovial cells have been found (48–50). The expression levels of both CXCL12 and CXCR4 were also shown to positively correlate with ESR, CRP, RF, and DAS28 scores (51). As there is a CXCL12-based chemoattraction in RA towards the inflamed joints, these Classical Monocytes turned Foam cells could be migrating to the site of joint inflammation, where they could contribute to the pathogenesis of RA.

In conclusion, TNF reception and the initiated response are greatly affected by the expression of both TNFR1 and TNFR2, with the latter taking up the most prominent role by forming the activation threshold for the response to TNF.

## Data Availability

The datasets presented in this study can be found in online repositories. The names of the repository/repositories and accession number(s) can be found below: https://www.ncbi.nlm.nih.gov/geo/, GSE289019.
