# Supplementary material for: Single-cell multi-omics reveals the TNF-α activation threshold for Classical Monocytes by studying healthy donors and rheumatoid arthritis patients
Source: Front Immunol. 2025 May 16;16:1572823. doi: 10.3389/fimmu.2025.1572823 (PMC12122427; doi:10.3389/fimmu.2025.1572823)
Supplement: Supplementary file 1 [file DataSheet1.docx]

Supplementary Material


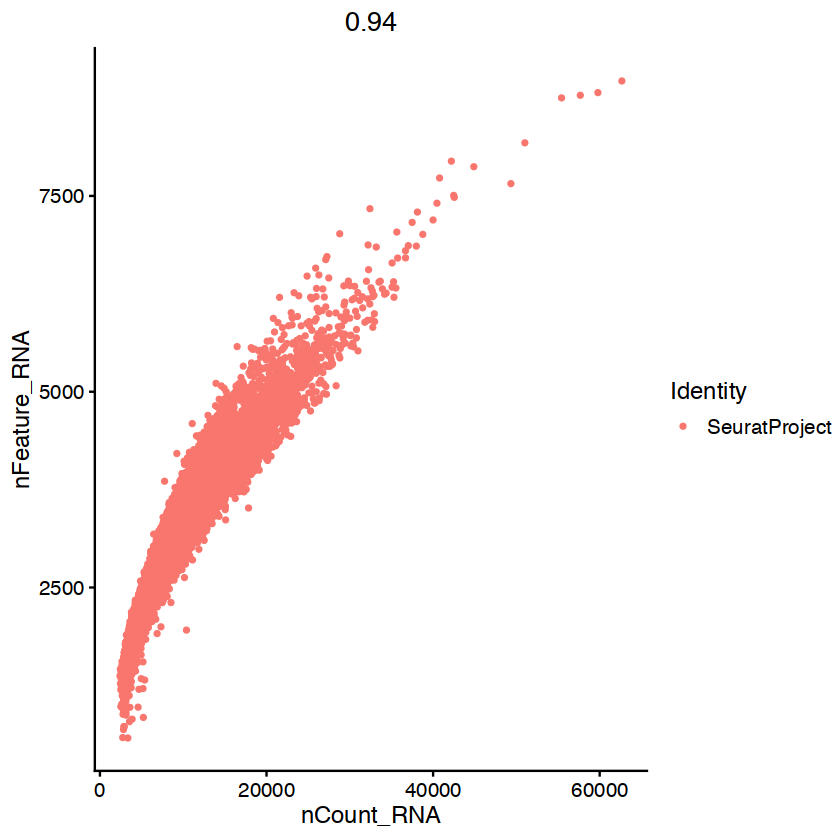


**Supplementary Figure 1**. Pre-QC gene expression dot plot.


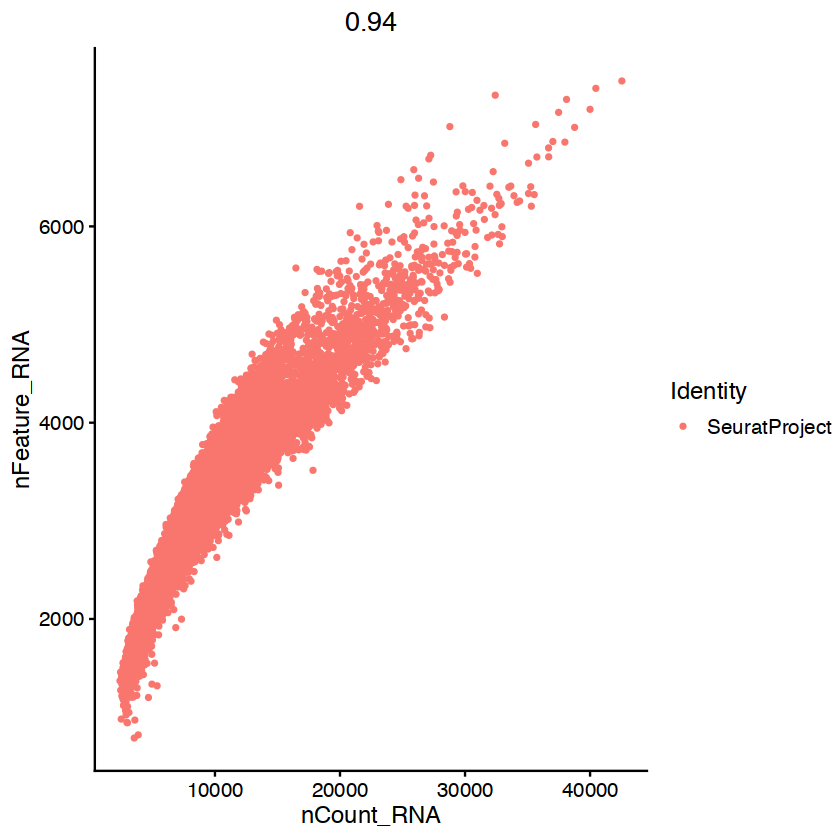


**Supplementary Figure 2**. Post-QC gene expression dot plot.


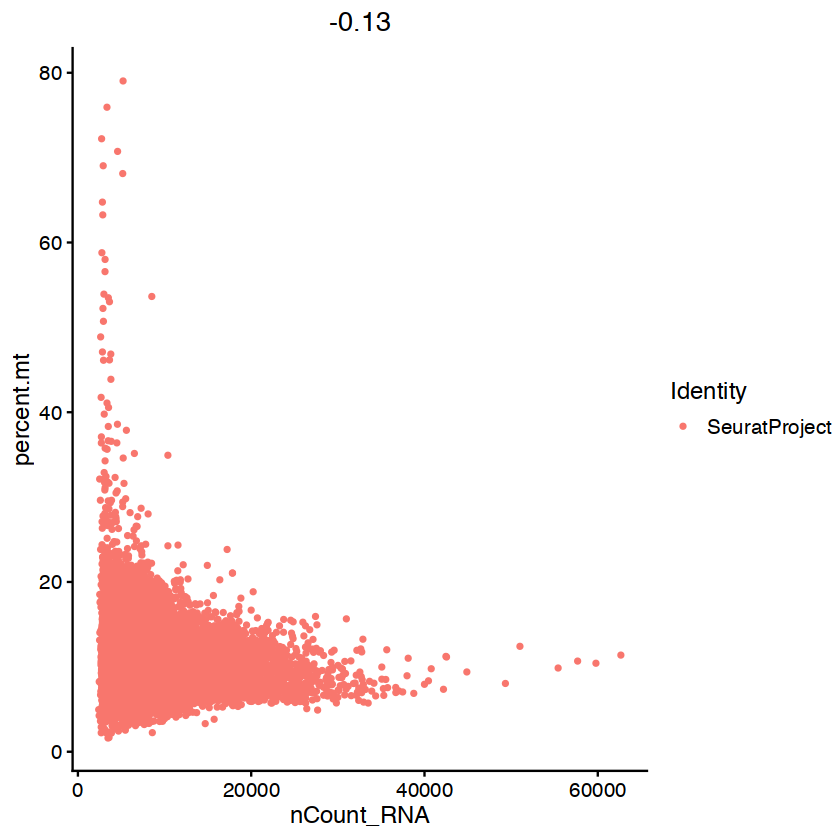


**Supplementary Figure 3**. Pre-QC gene expression vs percent.mt dot plot (BD Rhapsody uses stronger lysis buffers thus the higher percent.mt values).


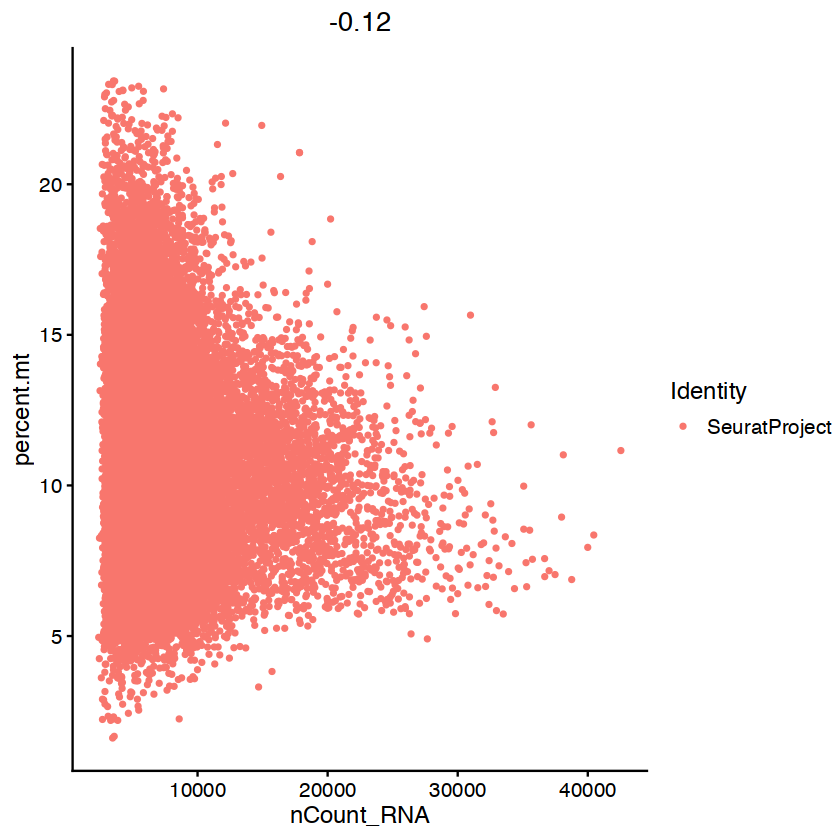


**Supplementary Figure 4**. Post-QC gene expression vs percent.mt dot plot (BD Rhapsody uses stronger lysis buffers thus the higher percent.mt values).


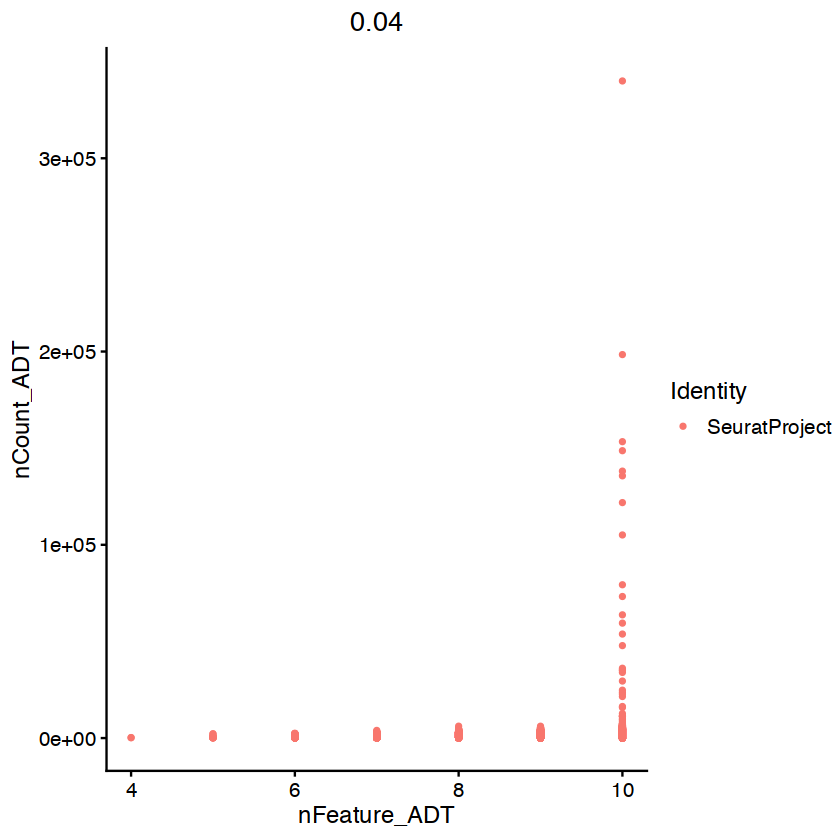


**Supplementary Figure 5**. Pre-QC protein expression dot plot.


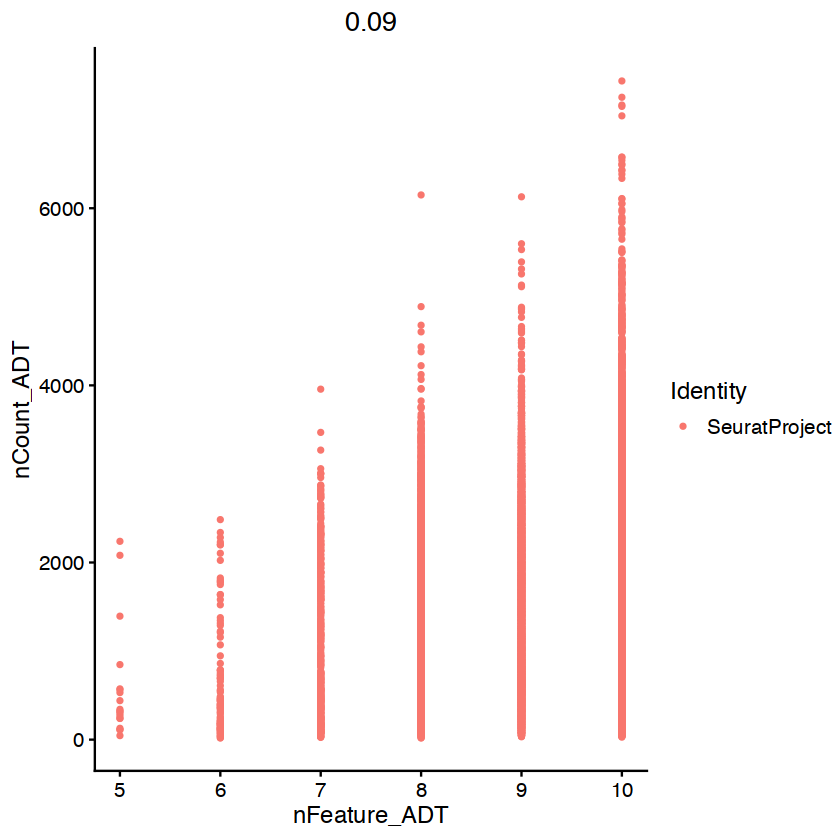


**Supplementary Figure 6**. Post-QC protein expression dot plot.


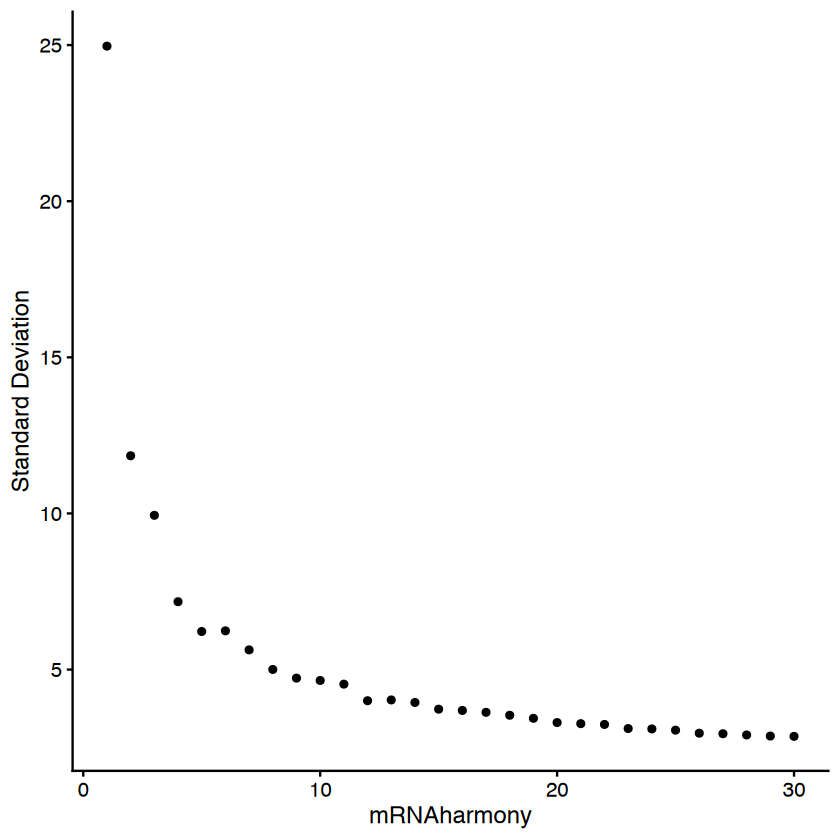


**Supplementary Figure 7**. SC-transformed and Harmony-corrected gene expression PC elbow plot.


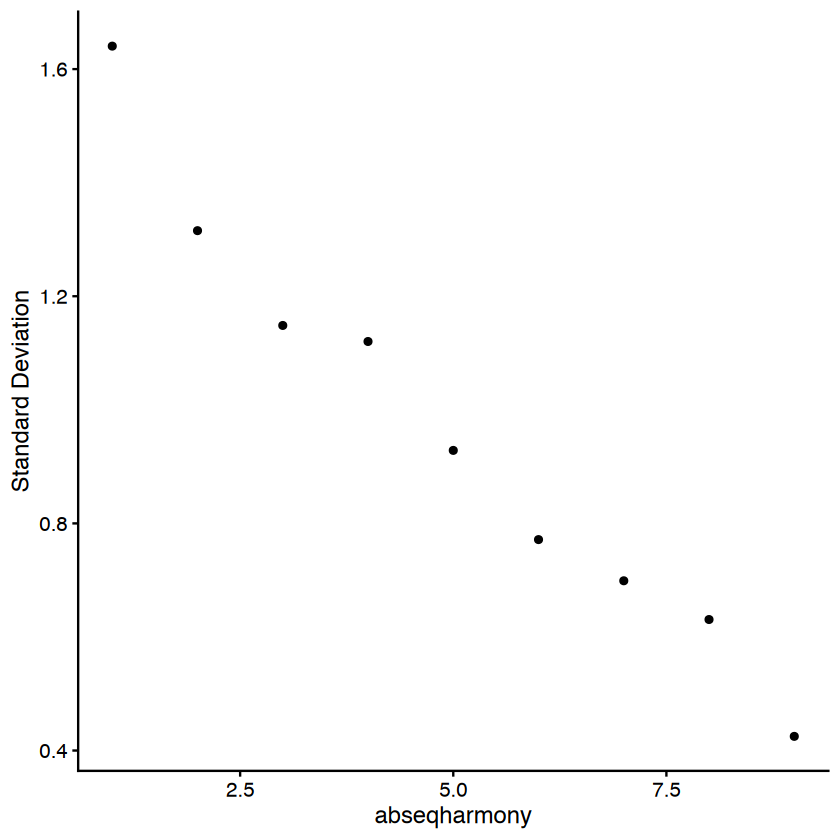


**Supplementary Figure 8**. CLR-transformed and Harmony-corrected protein expression PC elbow plot.


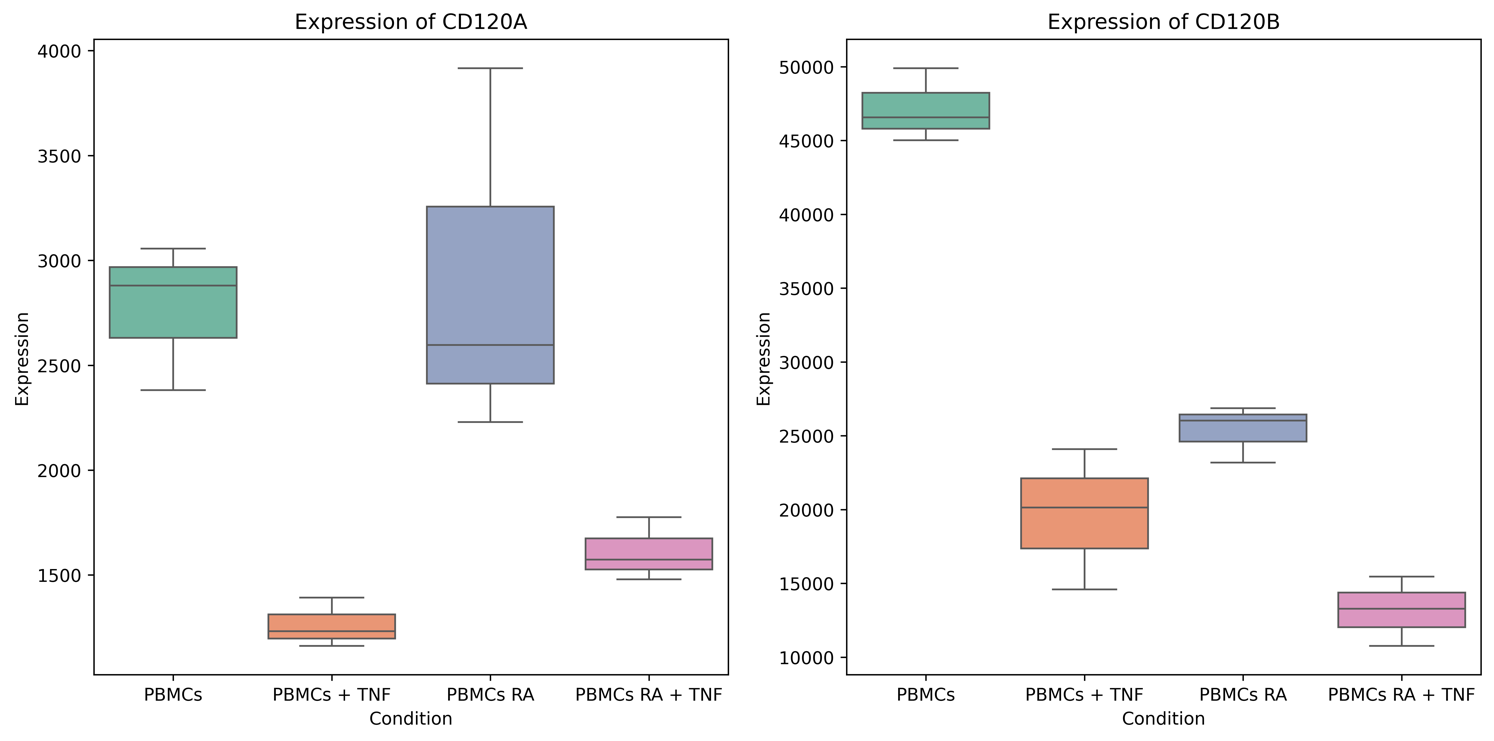


**Supplementary Figure 9.** Box plot of the TNFR1 (CD120A) and CD120B (TNFR2) protein expression.

| **Comparison** | **Protein** | **log2FoldChange** | ***q*-value** |
| --- | --- | --- | --- |
| PBMCs +TNF vs PBMCs | CD120A | -1.1438413423885982 | 7.268585514614103e-12 |
|  | CD120B | -1.306310524189259 | 8.318147883825118e-07 |
| PBMCs RA +TNF vs PBMCs RA | CD120A | -0.8821916548518607 | 0.04466170808720774 |
|  | CD120B | -0.9618751866766123 | 0.001073120145025014 |

**Supplementary Table 1.** DESeq2-derived log2(FC) and q-values.
